# Impact of tardive dyskinesia on patients and caregivers: a survey of caregivers in the United States

**DOI:** 10.1186/s41687-023-00658-9

**Published:** 2023-11-28

**Authors:** Rakesh Jain, Rajeev Ayyagari, Debbie Goldschmidt, Mo Zhou, Stacy Finkbeiner, Sam Leo

**Affiliations:** 1 Texas Tech University School of Medicine–Permian Basin, Midland, TX USA; 2 Analysis Group, Inc., Boston, MA USA; 3 Teva Branded Pharmaceutical Products R&D, Inc., North America Medical Affairs, Parsippany, NJ USA; 4 Teva Branded Pharmaceutical Products R&D, Inc., Global Health Economics and Outcomes Research, Parsippany, NJ USA

**Keywords:** Tardive dyskinesia, Caregiver burden, Patient burden, Work impact, Physical impact, Psychosocial impact

## Abstract

**Background:**

Tardive dyskinesia (TD) has a multidimensional impact on patients with TD and, as importantly, their caregivers. An online survey was developed and administered to assess patient and caregiver burden of TD. Survey participants were unpaid caregivers for patients with diagnoses of TD and schizophrenia, bipolar disorder, and/or major depressive disorder. Overall, 162 caregivers rated the 7-day impact of TD on the physical, psychological, and social functioning of patients and the impact of TD on these domains in their own lives and in their professional lives.

**Results:**

Across physical, psychological, and social domains, most caregivers (82.7%) reported that TD had severe impact on the cared-for patients, and 23.5% reported severe impact of TD in their own lives. Caregivers experienced 46.4% activity impairment, and caregivers who were employed (n = 136) experienced 49.5% overall work impairment because of TD-related caregiving.

**Conclusions:**

These results suggest that TD imposes substantial burden for both caregivers and patients.

**Supplementary Information:**

The online version contains supplementary material available at 10.1186/s41687-023-00658-9.

## Introduction

Tardive dyskinesia (TD) is an iatrogenic, hyperkinetic movement disorder with substantial impact on patients and caregivers [1, 2]. The prevalence of TD has increased from 6.88 per 100,000 patients in 2016 to 11.53 per 100,000 in 2020, which may be related to an aging population, increased use of antipsychotic agents, and improved clinician awareness of TD [1]. TD can occur subsequent to treatment with dopamine-receptor antagonists, including typical and atypical antipsychotic agents often used to treat schizophrenia (SCZ), bipolar disorder (BD), and major depressive disorder (MDD) [3]. Patients with TD experience abnormal involuntary movements that can impact activities of daily living, psychological and social functioning, and employment beyond the effects of their underlying psychiatric condition [4, 5].

As some patients with TD are unaware of their movements, exacerbated by deficits from their underlying psychiatric illness in some cases, caregiver reports can provide a more complete picture of patient burden from TD [6]. In addition, some patients with TD may not be able to participate in a survey because of the severity or location/topography of their involuntary movements (e.g., impaired motor function in upper limbs may limit ability to operate a computer, orofacial movements may limit ability to speak) [7], further supporting the role of caregiver-reported observations in providing a comprehensive view of patient burden related to TD.

A variety of treatment modalities have been investigated, since reports of dyskinetic movements related to antipsychotic agents have surfaced [8]. Vesicular monoamine transporter (VMAT) 2 inhibitors have substantial therapeutic evidence [9–11], and are recommended by the American Psychiatric Association (APA) for first-line treatment of moderate-to-severe TD symptoms. Other potential pharmacologic treatments include anticholinergics; however, anticholinergics do not improve, and may even worsen TD [12, 13], in addition to producing considerable side effects. There are some adjunct therapeutic options such as, electroconvulsive therapy [14], and dietary supplements such as vitamin E and B_6_ and essential fatty acids [15, 16], but evidence for their effectiveness is lacking, and therefore, they are not recommended by the APA.

Informal caregivers (e.g., unpaid family members, friends) are often a primary source of care for patients and caregivers’ physical and psychological health can be impacted by caregiving activities [17, 18], leading some to refer to caregivers as the “invisible patient” [17]. Caregivers play an important role in monitoring TD symptoms and treatment of TD. Caregivers are more likely to notice subtle symptoms and are in a position to monitor for potentially life-threatening complications of TD, such as dyspnea and dysphagia [19]. It is therefore important for clinicians to recognize and assess caregiver burden. Subjective caregiver burden is known to be a risk factor for anxiety [20] and in the recent RE-KINECT study, caregivers of patients with TD reported about the impact on their own ability to function and quality of life [21].

This survey-based study was designed to further assess and provide details about the experiences and concerns of caregivers of patients with TD in the United States, including self-reported burden of caregiving tasks, psychological well-being, daily activities, and professional life. As caregiver burden has been shown to correlate with caregiver perception of patient burden, the present study also reports caregiver-assessed impact of TD on physical, psychological, and social domains of the cared-for patient.

## Methods

### Study design

Online English-language surveys to assess the burden of TD were developed for patients and caregivers using a targeted literature review of existing publications on TD burden and one-on-one phone interviews with patients, caregivers, and health care providers. The study was approved by Western Institutional Review Board (IRB) and informed consent was obtained from interview participants and survey respondents before their involvement in the study. This report focuses on caregiver responses to the surveys; results for the parallel survey study conducted with an independent patient population are reported separately [22].

The initial online survey was reviewed and tested to verify the content accuracy and clarity, and real-time online quality checks were performed to ensure data coherence and logic. In addition, beta testing was conducted with 3 eligible caregivers and the online survey was modified based on their feedback. In the final survey, caregivers provided responses regarding the impact of TD on the cared-for patient’s physical, psychological, and social functioning, rated on a 5-point Likert scale, and the impact of TD-related caregiving tasks on the caregiver’s psychological well-being and daily activities. The impact of TD on caregivers’ professional lives and overall activity impairment was assessed using the Work Productivity and Activity Impairment Questionnaire (WPAI [23]) as well as several questions designed specifically for this study. All questions included “anchoring” language to isolate the impact of TD from the impact of the patient’s underlying condition. For example, the term “irregular movements” was added to the WPAI items to isolate the impact of TD from the impact of other comorbidities. For most questions, respondents were required to provide a response in order to proceed with the survey, limiting instances of missing data.

### Survey participants

Caregivers were recruited from general population panels maintained by the Schlesinger Group, a market research firm, and their partners. These panels included healthy participants (including caregivers) as well as patients with various health conditions across the US who have agreed to receive invitations to participate in web-based surveys. Caregiver participants were required to be providing unpaid care for ≥ 3 months to a patient with current diagnoses of TD and SCZ, BD, and/or MDD and who, to the caregiver’s knowledge, had not already participated in the patient portion of this study [22].

### Statistical analysis

Likert scale responses for each domain item were converted to scores from 1 (no impact) to 5 (most impact) and used to calculate the summary score for each domain. Proportions of caregivers reporting severe impact for the patient or themselves (score ≥ 4 on at least 1 item within each domain) and mean impact scores were calculated for each domain and summarized descriptively; the distributions of responses for each item within the domains are presented descriptively, along with work and activity impairment results. The definition of severe impact was pre-defined and modeled after the scoring method used for the validated Abnormal Involuntary Movement Scale [24]. Because an average score may mask/dilute important disease impacts, a severe impact on a single item within a domain has the potential to have severe impact on the entire domain (i.e., choking may have more impact on a patient’s life than more frequent but potentially less distressing clenching of the teeth). Subgroup analyses were conducted by severity of TD as reported by the caregiver (no/mild/moderate, severe/very severe) and by patient’s underlying condition (SCZ, BD, MDD). If only one underlying condition was present, patients were stratified by the underlying disease; if SCZ and BD and/or MDD were present, patients were stratified by the condition having the greatest impact; if the caregiver reported being unsure which condition had the greatest impact on their patient's life, the patient was classified as having SCZ, and patients with BD and MDD (without SCZ) were classified as having BD.

## Results

### Caregiver and caregiver–patient relationship characteristics

A total of 162 caregivers completed the survey. Because multiple recruitment sources were utilized, it was not possible to determine an accurate response rate. The mean (SD) age of caregivers was 40.0 (9.8) years; the mean (SD) age of cared-for patients was 62.6 (15.4) years. Caregivers represented the diversity of race/ethnicities present in the United States [25] (76.5% White or Caucasian; 19.1% Latino, Hispanic, or Chicano; 10.5% Black or African American; 5.6% Asian); however, caregivers with higher education may have been overrepresented [25] (Table 1). Most caregivers reported that the patient was their parent or guardian (56.2%) and that they had been caring for them for 1–‍10 years (80.9%). Caregivers reported that 21.0% of cared-for patients were on long-term disability, and 54.3% were retired (Table 1).

**Table 1:** Key caregiver and patient demographic characteristics

	Caregivers of patients…				Patients (as reported by caregivers)…			
	Overall N = 162	With SCZ n = 47	With BD n = 67	With MDD n = 48	Overall N = 162	With SCZ n = 47	With BD n = 67	With MDD n = 48
Age, years, mean (SD)	40.0 (9.8)	38.9 (10.1)	39.7 (8.9)	41.1 (10.7)	62.6 (15.4)	62.9 (15.3)	60.2 (15.6)	65.6 (14.8)
Male, n (%)	106 (65.4)	27 (57.4)	46 (68.7)	33 (68.8)	87 (53.7)	24 (51.1)	36 (53.7)	27 (56.3)
Race/ethnicity,^a^ n (%)								
White or Caucasian	124 (76.5)	35 (74.5)	50 (74.6)	39 (81.3)	126 (77.8)	34 (72.3)	52 (77.6)	40 (83.3)
Latino, Hispanic, or Chicano	31 (19.1)	10 (21.3)	15 (22.4)	6 (12.5)	30 (18.5)	10 (21.3)	14 (20.9)	6 (12.5)
Black or African American	17 (10.5)	6 (12.8)	6 (9.0)	5 (10.4)	16 (9.9)	7 (14.9)	5 (7.5)	4 (8.3)
Asian	9 (5.6)	4 (8.5)	3 (4.5)	2 (4.2)	9 (5.6)	4 (8.5)	3 (4.5)	2 (4.2)
Native Hawaiian or Pacific Islander	1 (0.6)	0	1 (1.5)	0	1 (0.6)	0	1 (1.5)	0
Other racial/ethnic background	1 (0.6)	0	0	1 (2.1)	1 (0.6)	1 (2.1)	0	0
Education, n (%)								
High school diploma or equivalent	10 (6.2)	3 (6.4)	5 (7.5)	2 (4.2)	35 (21.6)	5 (10.6)	17 (25.4)	9 (18.8)
Some college, no degree	13 (8.0)	3 (6.4)	6 (9.0)	4 (8.3)	23 (14.2)	8 (17.0)	9 (13.4)	6 (12.5)
Associate’s degree	18 (11.1)	4 (8.5)	7 (10.4)	7 (14.6)	19 (11.7)	6 (12.8)	7 (10.4)	6 (12.5)
Bachelor’s degree	69 (42.6)	24 (51.1)	30 (44.8)	15 (31.3)	64 (39.5)	20 (42.6)	24 (35.8)	20 (41.7)
Graduate degree	52 (32.1)	13 (27.7)	19 (28.4)	20 (41.7)	21 (13.0)	6 (12.8)	8 (11.9)	7 (14.6)
Employment status, n (%)								
Currently employed	136 (84.0)	41 (87.2)	55 (82.1)	40 (83.3)	8 (4.9)	1 (2.1)	6 (9.0)	1 (2.1)
Working full-time	107 (66.0)	31 (66.0)	44 (65.7)	32 (66.7)	3 (1.9)	0	2 (3.0)	1 (2.1)
Working part-time	22 (13.6)	9 (19.1)	6 (9.0)	7 (14.6)	5 (3.1)	1 (2.1)	4.6 (6.0)	0
Self-employed	8 (4.9)	1 (2.1)	6 (9.0)	1 (2.1)	0	0	0	0
On short-term disability from work	1 (0.6)	0	0	1 (2.1)	5 (3.1)	2 (4.3)	1 (1.5)	2 (4.2)
On long-term disability from work	3 (1.9)	0	2 (3.0)	1 (2.1)	34 (21.0)	12 (25.5)	16 (23.9)	6 (12.5)
Not employed, looking for work	6 (3.7)	1 (2.1)	4 (6.0)	1 (2.1)	4 (2.5)	0	4 (6.0)	0
Not employed, not looking for work	3 (1.9)	1 (2.1)	2 (3.0)	0	22 (13.6)	6 (12.8)	7 (10.4)	9 (18.8)
Retired	7 (4.3)	2 (4.3)	2 (3.0)	3 (6.3)	88 (54.3)	24 (51.1)	33 (49.3)	31 (64.6)
Student	1 (0.6)	1 (2.1)	0	0	1 (0.6)	1 (2.1)	0	0
Homemaker	4 (2.5)	0	2 (3.0)	2 (4.2)	4 (2.5)	1 (2.1)	3 (4.5)	0
Other	2 (1.2)	1 (2.1)	1 (1.5)	0	0	0	0	0

*BD* Bipolar disorder, *MDD* Major depressive disorder, *SCZ* Schizophrenia

^a^Respondents could select more than 1 option; results are not mutually exclusive

Most patients had a primary underlying psychiatric condition of BD (n = 67, 41.4%), followed by MDD (n = 48, 29.6%) and SCZ (n = 47, 29.0%). Despite 90.7% of cared-for patients currently receiving TD medication, 35.2% of caregivers reported that the patient’s TD symptoms were severe or very severe, and 69.8% described patients as being either quite a bit or very much bothered by their TD symptoms (Table 2).

**Table 2:** Key caregiver–patient relationship and patient clinical characteristics

	Caregivers of patients…			
	Overall N = 162	With SCZ n = 47	With BD n = 67	With MDD n = 48
Relationship to patient, n (%)				
Patient is parent or guardian	91 (56.2)	23 (48.9)	38 (56.7)	30 (62.5)
Spouse or partner	19 (11.7)	7 (14.9)	9 (13.4)	3 (6.3)
Sibling	11 (6.8)	4 (8.5)	3 (4.5)	4 (8.3)
Friend	6 (3.7)	4 (8.5)	0	2 (4.2)
Patient is my child	2 (1.2)	1 (2.1)	1 (1.5)	0
Other relative	33 (20.4)	8 (17.0)	16 (23.9)	9 (18.8)
Living with patient, n (%)	96 (59.3)	29 (61.7)	35 (52.2)	32 (66.7)
Length of time as caregiver, n (%)				
≥ 3 months to ≤ 1 year	22 (13.6)	6 (12.8)	7 (10.4)	9 (18.8)
> 1 year to ≤ 3 years	78 (48.1)	25 (53.2)	34 (50.7)	19 (39.6)
> 3 years to ≤ 10 years	53 (32.7)	12 (25.5)	24 (35.8)	17 (35.4)
> 10 years	9 (5.6)	4 (8.5)	2 (3.0)	3 (6.3)
Patient currently receiving TD medications, n (%)	147 (90.7)	45 (95.7)	60 (89.6)	42 (87.5)
Severity of patient’s TD symptoms in previous 7 days, n (%)				
None	2 (1.2)	1 (2.1)	1 (1.5)	0
Mild	9 (5.6)	3 (6.4)	2 (3.0)	4 (8.3)
Moderate	94 (58.0)	24 (51.1)	40 (59.7)	30 (62.5)
Severe	47 (29.0)	16 (34.0)	19 (28.4)	12 (25.0)
Very severe	10 (6.2)	3 (6.4)	5 (7.5)	2 (4.2)
Extent to which patient was bothered by TD symptoms in previous 7 days, n (%)				
Not at all	1 (0.6)	1 (2.1)	0	0
A little bit	12 (7.4)	2 (4.3)	8 (11.9)	2 (4.2)
Somewhat	36 (22.2)	10 (21.3)	11 (16.4)	15 (31.3)
Quite a bit	83 (51.2)	23 (48.9)	36 (53.7)	24 (50.0)
Very much	30 (18.5)	11 (23.4)	12 (17.9)	7 (14.6)

*BD* Bipolar disorder, *MDD* Major depressive disorder, *SCZ* Schizophrenia, *TD* Tardive dyskinesia

### Caregiver-reported impact of TD on patients

**Fig. 1:**
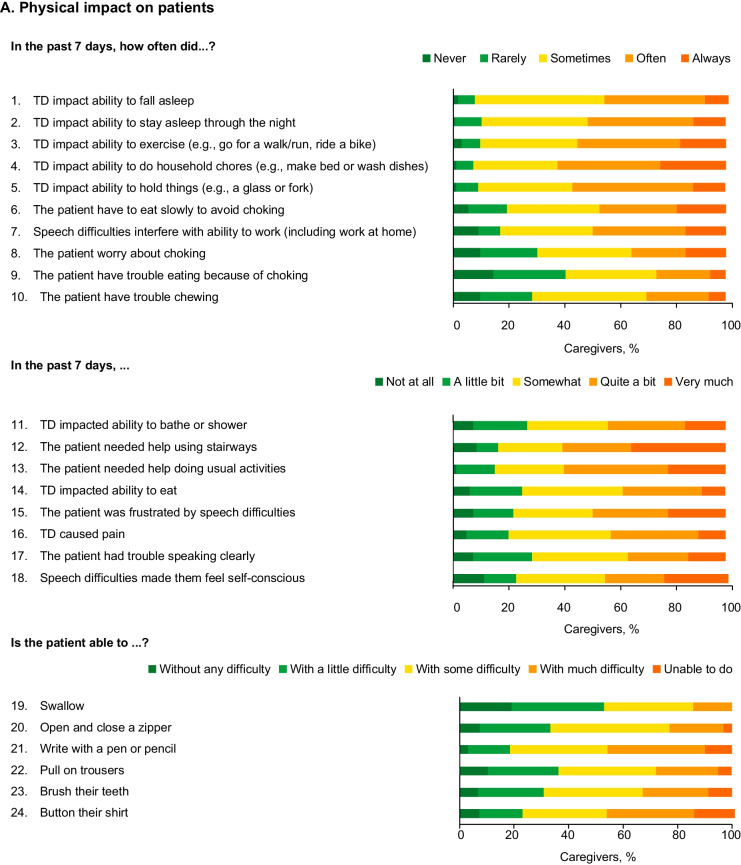

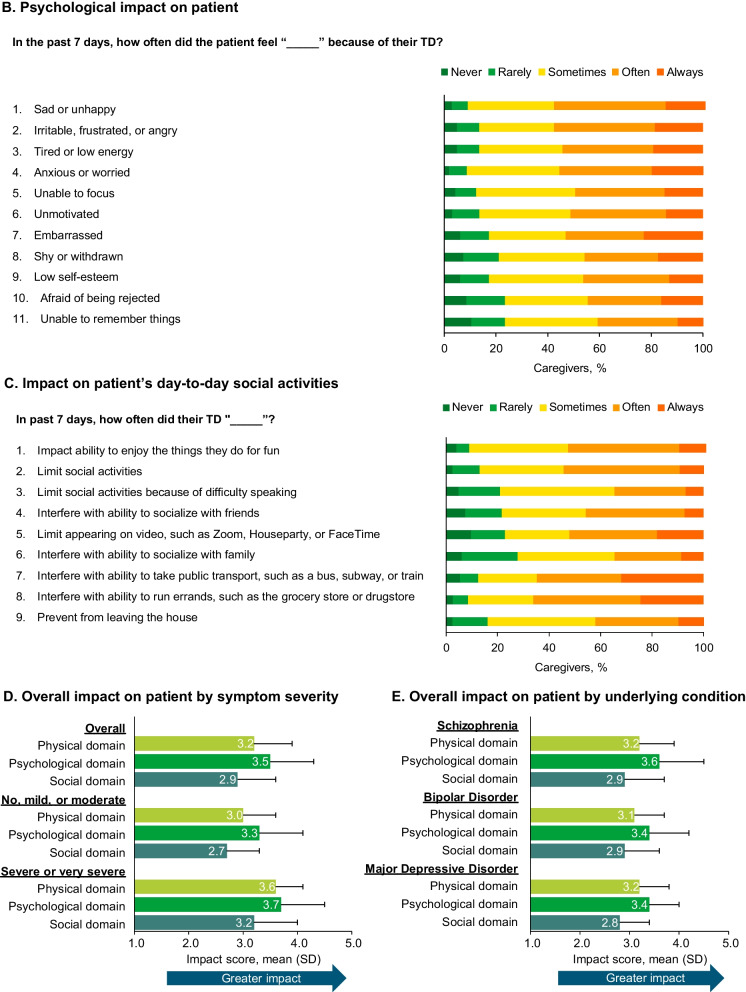
Caregiver-reported impact of TD on patients. *TD* Tardive dyskinesia

Most (82.7%) caregivers reported that TD had a severe impact (impact score ≥ 4 on at least 1 item within each domain) on the cared-for patient across physical, psychological, and social domains. On a scale from 1 to 5 (with 1 being no impact and 5 being most impact), the mean (SD) physical impact score reported by caregivers was 3.2 (0.7) and increased with TD symptom severity (3.0 [0.6] for no/mild/moderate versus 3.6 [0.5] for severe/very severe) (Fig. 1D).

The mean (SD) scores for physical impact of TD on patients were reported by caregivers to be similar across underlying disease conditions (SCZ, 3.2 [0.7]; BD, 3.1 [0.6]; MDD, 3.2 [0.6]) (Fig. 1E). Over 90% of caregivers reported that TD had moderate-to-severe impact on the cared-for patient’s sleeping habits, and ability to do exercise and household chores. More than 80% of caregivers reported moderate-to-severe impact of TD on the patient’s independence and work ability (Fig. 1A).

The mean (SD) caregiver-reported score for the psychological impact of TD on patients was 3.5 (0.8) and increased with reported severity of TD symptoms (3.3 [0.8] for no/mild/moderate versus 3.7 [0.8] for severe/very severe) (Fig. 1D). Caregivers reported greater psychological impact for patients with underlying SCZ (3.6 [0.9]) than for those with BD (3.4 [0.8]) or MDD (3.4 [0.6]) (Fig. 1E). Over 80% of caregivers reported that the cared-for patient sometimes/often/always felt frustrated, sad, worried, unmotivated and embarrassed (Fig. 1B).

The mean (SD) caregiver-reported score for the social impact of TD on patients was 2.9 (0.7), and also increased with reported TD severity (2.7 [0.6] for no/mild/moderate versus 3.2 [0.8] for severe/very severe) (Fig. 1D). Over three-quarters of caregivers reported that TD sometimes/often/always impacted the ability of the patients to socialize remotely or in person with other people, including friends, as well as difficulty leaving the house (Fig. 1C). Over half of caregivers reported that acquaintances sometimes/often/always stared or looked at the patient and asked about their abnormal movements (Additional file 1: Fig. S1).

### Caregiver burden

Mean (SD) impact scores were 2.1 (1.1) for TD-related caregiving tasks, 2.5 (0.9) for caregiver psychological well-being, and 2.7 (0.9) for caregiver daily activities, each increasing with TD symptom severity. For caregivers of patients with no/mild/moderate symptoms, scores were 2.0 (1.0), 2.3 (0.9), and 2.6 (0.9), respectively, and for caregivers of patients with severe/very severe TD symptoms, scores were 2.4 (1.2), 2.7 (1.0), and 2.9 (1.0). Nearly 1 in 4 caregivers (23.5%) reported severe impact across all 3 domains; proportions were greater for caregivers of patients with severe/very severe TD symptoms (33.3%) than those with no/mild/moderate TD symptoms (18.1%), as well as for caregivers of patients with BD (25.4%) or MDD (25.0%) compared with those with SCZ (19.1%).

**Fig. 2:**
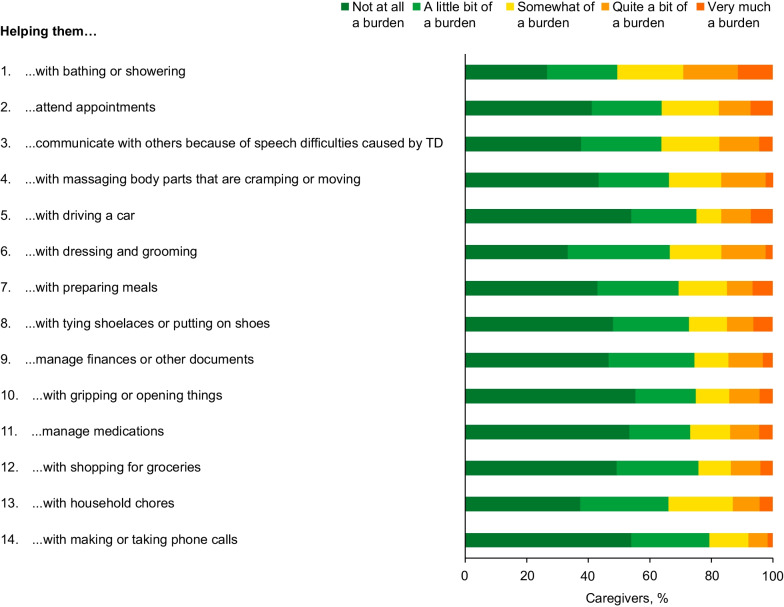
Burden of TD-related caregiving tasks. *TD* Tardive dyskinesia. Respondents only provided the burden of tasks for which they indicated they support the patient

Shopping for groceries (76.5%), preparing meals (74.7%), managing medications (71.6%), doing household chores (71.0%), and driving a car (69.8%) were the most common tasks for which caregivers provided assistance. The caregiving task reported as most burdensome was helping patients with bathing or showering; however, the majority of caregivers did not consider helping patients with any individual task burdensome (Fig. 2). While 44.4% of caregivers reported that none of the tasks they helped patients with were somewhat, quite a bit, or very much of a burden, proportions of caregivers who reported that the tasks were somewhat, quite a bit, or very much of a burden for the cared-for patient were lower for those with severe/very severe TD symptoms (36.8%) compared with no/mild/moderate symptoms (48.6%), as well as for patients with MDD (39.6%) or BD (40.3%) compared with SCZ (55.3%) (Additional file 2: Fig. S2).

**Fig. 3:**
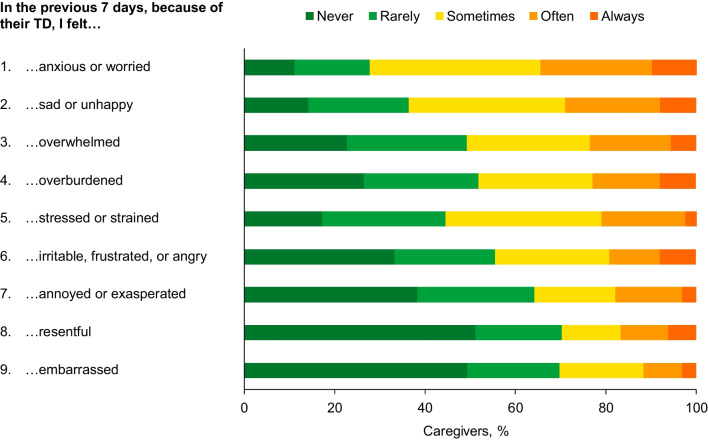
Impact on caregiver psychological well-being. *TD* Tardive dyskinesia

Over one-third of caregivers (34.6%) reported often or always feeling anxious or worried because of the patient’s TD. Moreover, 29.0% reported often/always feeling sad or unhappy, 23.5% overwhelmed, 22.8% overburdened, and 21.0% stressed or strained (Fig. 3). Although 17.3% of caregivers reported experiencing all 9 of these negative emotions at least sometimes, the proportion was higher among caregivers of patients with severe/very severe TD symptoms (24.6%) than for those with no/mild/moderate TD symptoms (13.3%). No clear trend was observed for patients’ underlying conditions (Additional file 3: Fig. S3).

**Fig. 4:**
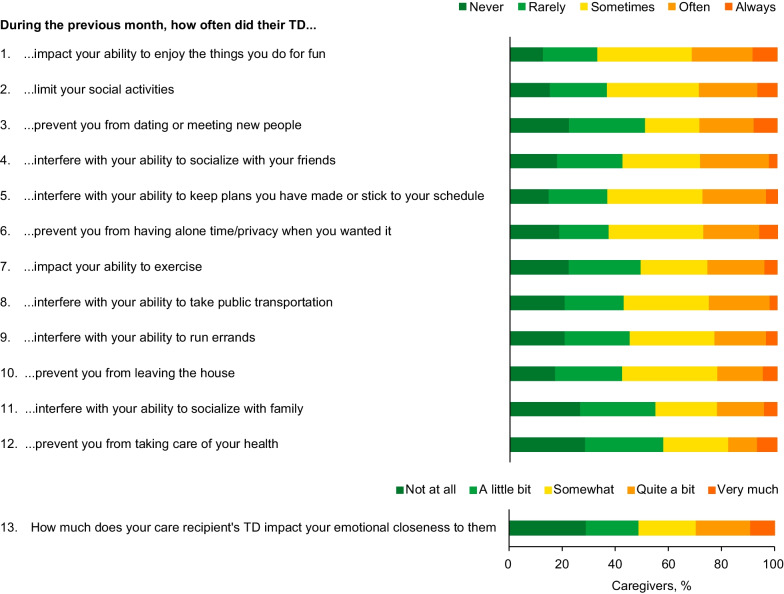
Impact on caregiver daily activities. *TD* Tardive dyskinesia

Approximately 30% of caregivers reported that their ability to enjoy the things they do for fun, to join social activities, to socialize with friends, and/or to date or meet new people was often or always impacted by the patient’s TD, with greater impact for caregivers of patients with more severe TD (Fig. 4). In addition, 29.7% of caregivers reported that the patient’s TD impacted their emotional closeness with the patient quite a bit or very much. Some caregivers (8.6%) reported that the patient’s TD impacted all 13 of the included activities at least sometimes, with greater proportions for caregivers of patients with severe/very severe TD symptoms (10.5%) than no/mild/moderate TD symptoms (7.6%); no clear trend was observed for patient’s underlying condition (Additional file 4: Fig. S4).

**Fig. 5:**
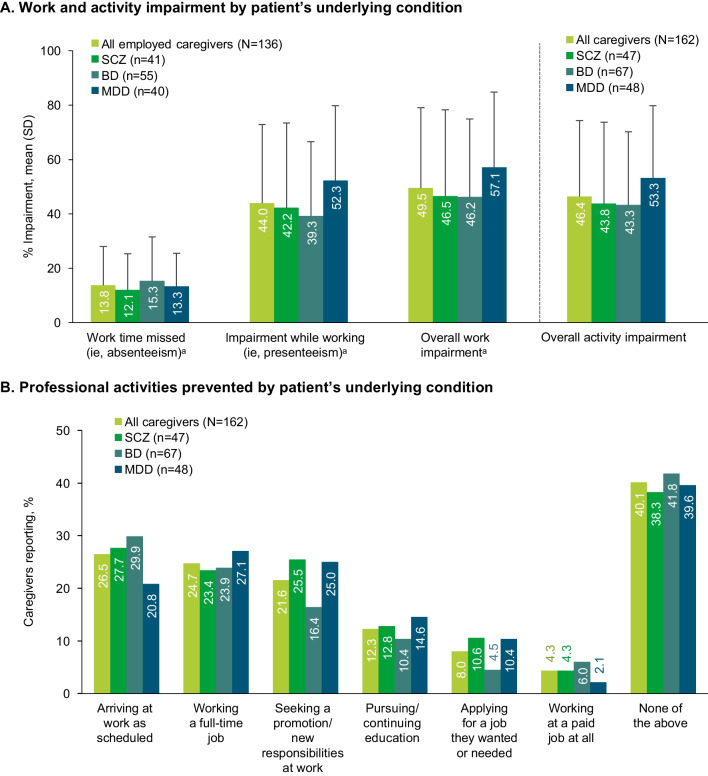
Impact on caregiver professional life. *BD* Bipolar disorder, *MDD* Major depressive disorder, *SCZ* Schizophrenia. Patients for whom caregivers were providing care were stratified by the underlying disease if only 1 condition was present or, if > 1 was present, by the condition having the greatest impact. Patients for whom caregivers were providing care with BD + MDD were classified as BD. ^a^Among caregivers who were employed

Based on WPAI responses, caregivers experienced 46.4% activity impairment (calculated as degree to which the problem affected regular activities [e.g., housework, shopping] rated from 0 [no effect] to 10 [completely prevented] × 10 [23]) because of TD-related caregiving tasks; caregivers for patients with MDD had the highest level of activity impairment (53.3%) compared with caregivers of patients with BD (43.3%) and SCZ (43.8%) (Fig. 5A).

Caregivers who were employed (n = 136, 84.0%; Table 1) missed 13.8% of work time (absenteeism calculated as missed work hours/number of scheduled work hours × 100 [23]) and experienced 44.0% impairment while working (presenteeism calculated as the degree to which the problem affected work productivity rated from 0 [no effect] to 10 [completely prevented] × 10 [23]), representing 49.5% overall work impairment (calculated as number of hours worked/number of scheduled work hours × presenteeism [13]). Caregivers for patients with MDD had the highest levels of presenteeism and overall work impairment (52.3% and 57.1%, respectively) compared with caregivers of patients with BD (39.3% and 46.2%) and SCZ (42.2% and 46.5%) (Fig. 5A). Most employed caregivers reported that caring for patients with TD limited them at work sometimes (38.2%), often (15.4%), or always (2.9%). Caring for patients with TD prevented approximately one-quarter of caregivers from working a full-time job or arriving at work as scheduled (Fig. 5B).

## Discussion

The results of the present survey provide an important lens on TD burden in patients as described by caregivers. In addition, this survey reports new descriptive data on the burden of TD caregiving tasks and on the impact of TD on caregiver daily activities, psychological well-being, and social life.

The mean age of patients for whom caregivers reported impact in this survey was 62.6 years. The severity of TD increases with age (up to approximately age 70), as does the mental impact [26, 27]. In addition, a large proportion of caregivers (82.7%) reported that TD had a severe impact across physical, psychological, and social domains, which supports the increasing age-related severity of TD. Over 90% of caregivers in this survey reported moderate-to-severe impact on patient’s sleep, over 80% reported patient sadness and anxiety, and over 75% reported interference in many aspects of the patient’s social life. This indicates a substantial impact of TD on this population of cared-for patients with 21% of patients reported to be on long-term disability.

While most caregivers did not report finding TD-related caregiving tasks to be burdensome (perhaps in part because of an unwillingness to admit it, even in an anonymous survey), caregiving did impact their psychological well-being, daily activities, and professional life. Over one-third of caregivers reported often or always feeling anxious or worried because of the patient’s TD. Previous studies of caregivers of patients with SCZ, BD, or MDD without TD revealed substantial impact of these conditions on caregiver burden [28–32]. Increased burden for caregivers of patients with essential tremor has been correlated with caregiver assessment of the patient’s embarrassment [33] and of the patient’s overall suffering [34]. This is consistent with the results of the recent RE-KINECT study that reported that caregivers’ perceptions of the involuntary movements of patients with TD impacted the caregivers’ own ability to continue usual activities, be productive, socialize, or perform self-care activities [21]. However, as the present study used a unique survey to assess burden, direct comparisons with other caregiver burden measures are difficult.

This study reports greater overall impact of TD on caregivers’ work presenteeism (44%) and overall work impairment (50%) than reported for caregivers of patients with Alzheimer’s disease (presenteeism, 33%; overall work impairment, 40%) [35] or with non-small cell lung cancer (presenteeism, 21%; overall work impairment: 26%) [36]. Caregivers of patients with TD and SCZ also reported greater impact on work absenteeism (12%) and presenteeism (42%) than previously reported for caregivers of patients with SCZ with unknown TD status (absenteeism, 5%; presenteeism, 25%) [37]. Likewise, caregivers of patients with TD and MDD reported higher levels of presenteeism (52%) and overall work impairment (57%) than previously reported for caregivers of patients with MDD without suicidal ideation (presenteeism, 35%; overall work impairment, 40%) [30]. Caregivers in this study also reported 46% overall activity impairment, with impairments of 44%, 43%, and 53% for caregivers of patients with TD and underlying SCZ, BD, and MDD, respectively. The overall activity impairment observed here for caregivers of patients with TD and MDD (53%) was greater than that recently reported for caregivers of patients with unknown TD status with MDD alone (40%), or MDD and suicidal ideation (49%) [30]. For each of the cited studies work impairment was assessed via WPAI, which is a standardized questionnaire for assessing work and activity impairment. However, it should be noted that these studies used an unmodified version of the WPAI, whereas “anchoring” language was added to the version of the WPAI utilized in this caregiver study to isolate the impact of TD from other underlying disorders. Thus, the comparisons with previous caregiver studies and underlying psychiatric disorders should be interpreted with caution.

### Limitations

As with any survey-based study, these results are subject to recall bias, selection bias, and non-random missing data (i.e., specifically omitting a particular answer option across questions). The online format allowed for a large sample size and anonymity; however, the caregivers included in this study may not be representative of the general TD caregiver population. Caregivers who participated in this study were more highly educated and/or may have been more computer literate than the general population and were potentially more informed/aware of TD, more motivated to participate in this study, and/or providing care for patients with more severe TD symptoms, as participation required recognition of TD. Caregivers may perceive impact of TD on patients differently than the patients themselves. However, patient-reported severity of abnormal movements in TD was shown to correlate with caregiver-reported severity in the recent RE-KINECT study [21]. While survey questions were written to ask specifically about the impact of TD (i.e., anchoring), caregivers may have had difficulty separating the impact of TD from that imposed by underlying conditions or other comorbidities. This survey focused exclusively on caregivers of patients with TD and SCZ, BD, or MDD; no control comparison was made with caregivers of patients with SCZ, BD, or MDD without TD. Additionally, the survey was conducted during the coronavirus 2019 pandemic, which may have influenced caregivers’ experiences and activity levels.

## Conclusions

This population of patients who relied on caregiver assistance experienced substantial impact of TD on physical, psychological, and social well-being according to caregiver report. Caregiver input may be an important resource for health care providers in assessing the impact of TD on the patient. Although most caregivers did not consider helping patients with individual tasks to be burdensome, responses regarding impact on their own activities and psychological well-being reflect a cumulative burden of supporting patients with TD. As subjective caregiver burden is known to be a risk factor for anxiety in unpaid caregivers, these results highlight the importance of recognizing the incremental negative impact of TD on caregivers and may help health care providers develop assessment tools for holistic evaluation of the caregiver burden in TD. These results also demonstrate that TD imposes a substantial burden on caregivers’ professional lives, which should be taken into account when considering the societal burden of TD.

### Supplementary Information

**Additional file 1: Figure S1.** Caregiver-reported reactions of others to patients with TD.

**Additional file 2: Figure S2.** Burden of caregiving tasks by patient’s underlying condition.

**Additional file 3: Figure S3.** Impact on caregiver psychosocial well-being by patient’s underlying condition.

**Additional file 4: Figure S4.** Impact on caregiver daily activities by patient’s underlying condition.

## Data Availability

Qualified researchers may request access to patient level data and related study documents, including the study protocol and the statistical analysis plan. Requests will be reviewed for scientific merit, product approval status, and conflicts of interest. Patient level data will be de-identified, and study documents will be redacted to protect the privacy of trial participants and to protect commercially confidential information. Please email USMedInfo@tevapharm.com to make your request.

## Abbreviations

BD: Bipolar disorder
IRB: Institutional Review Board
MDD: Major depressive disorder
SCZ: Schizophrenia
TD: Tardive dyskinesia
WPAI: Work Productivity and Activity Impairment Questionnaire
