# Correction: Impact of tardive dyskinesia on patients and caregivers: a survey of caregivers in the United States

**DOI:** 10.1186/s41687-024-00728-6

**Published:** 2024-05-17

**Authors:** Rakesh Jain, Rajeev Ayyagari, Debbie Goldschmidt, Mo Zhou, Stacy Finkbeiner, Sam Leo

**Affiliations:** 1 Texas Tech University School of Medicine–Permian Basin, Midland, TX USA; 2 Analysis Group, Inc, Boston, MA USA; 3 Teva Branded Pharmaceutical Products R&D, Inc, North America Medical Affairs, Parsippany, NJ USA; 4 Teva Branded Pharmaceutical Products R&D, Inc, Global Health Economics and Outcomes Research, Parsippany, NJ USA

**Correction: J Patient Rep Outcomes 7, 122 (2023)**. 10.1186/s41687-023-00658-9

Following publication of the original article, the authors reported that the reference in the second sentence of the Introduction cites the wrong article. Reference 1 cites the correct article in other instances in the article.

The updated text with the correct reference should read as follows:

The prevalence based on real-world diagnoses rates of TD has increased from 6.88 per 100,000 patients in 2016 to 11.53 per 100,000 in 2020, which may be related to an aging population, increased use of antipsychotic agents, and improved clinician awareness of TD [1].
