# Supplementary material for: Impact of tardive dyskinesia on patients and caregivers: a survey of caregivers in the United States
Source: J Patient Rep Outcomes. 2023 Nov 28;7:122. doi: 10.1186/s41687-023-00658-9 (PMC10684842; doi:10.1186/s41687-023-00658-9)
Supplement: Supplementary file 1 — Additional file 1: Figure S1. Caregiver-reported reactions of others to patients with TD. [file 41687_2023_658_MOESM1_ESM.pdf]

**Figure S1. Caregiver-reported reactions of others to patients with TD**

**How often do people that they know (such as family, friends, or coworkers) "_____"?**

1. Stare or look at them because of TD
2. Ask what is wrong or why they are moving
3. Tell them to stop moving
4. Make jokes at their expense because of TD
5. Tell them that they are different than how they used to be because of TD
6. Grab or touch them to try to stop them from moving
7. Tell them that other people are looking at them because of TD

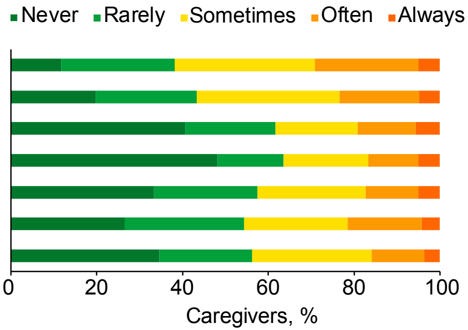


*TD* tardive dyskinesia
