# Supplementary material for: Impact of tardive dyskinesia on patients and caregivers: a survey of caregivers in the United States
Source: J Patient Rep Outcomes. 2023 Nov 28;7:122. doi: 10.1186/s41687-023-00658-9 (PMC10684842; doi:10.1186/s41687-023-00658-9)
Supplement: Supplementary file 2 — Additional file 2: Figure S2. Burden of caregiving tasks by patient’s underlying condition. [file 41687_2023_658_MOESM2_ESM.pdf]

Figure S2. Burden of caregiving tasks by patient's underlying condition

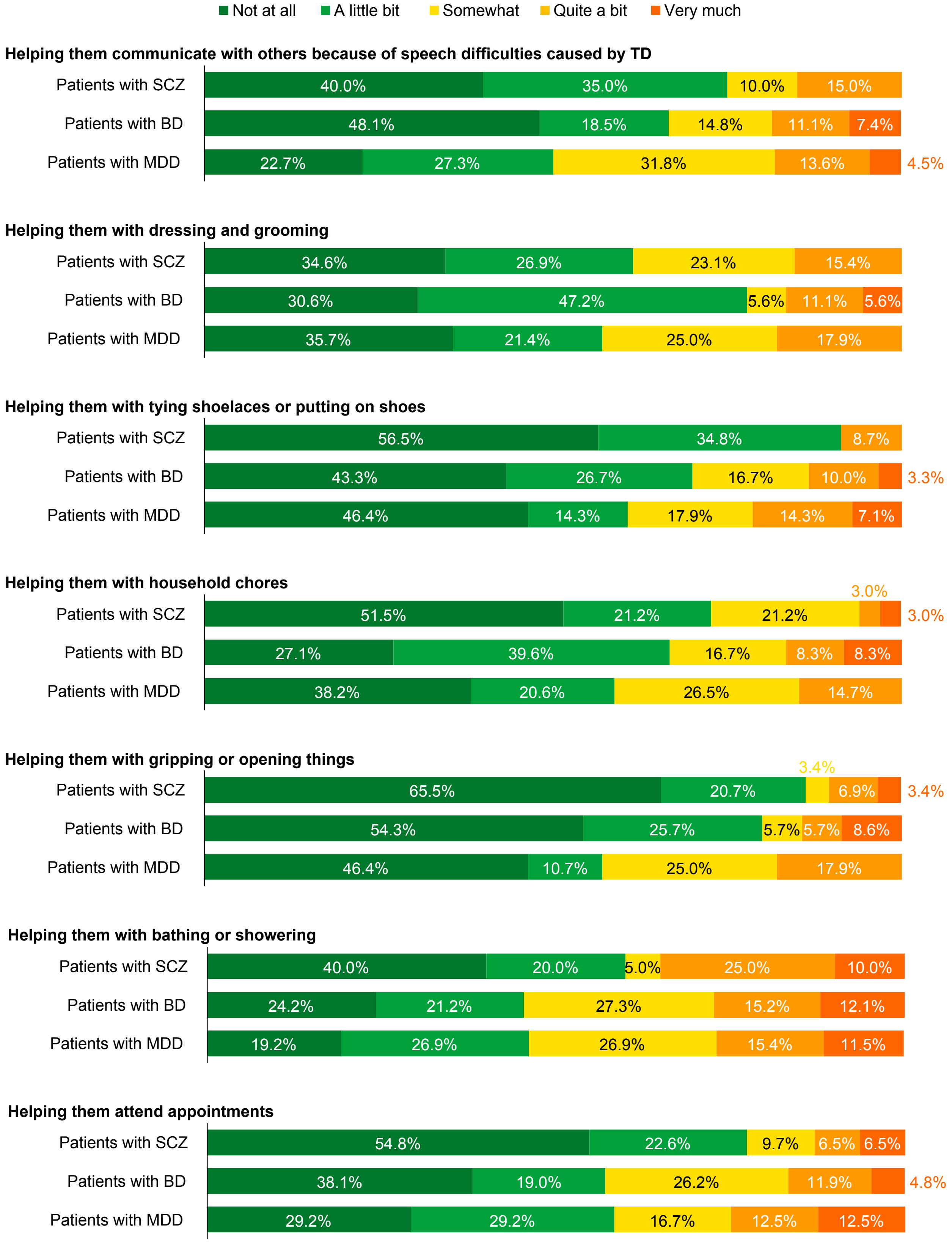

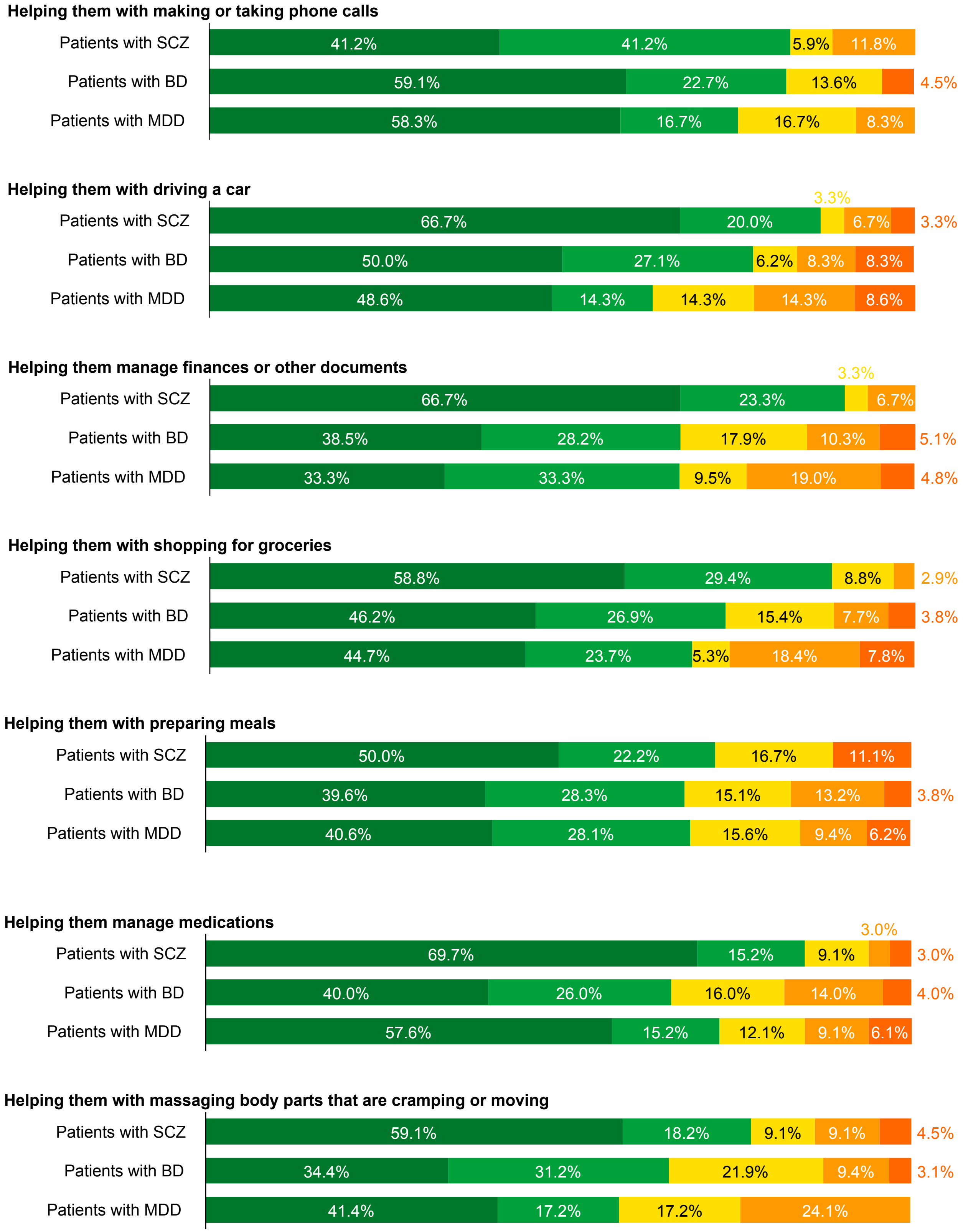

BD bipolar disorder, *MDD* major depressive disorder, *SCZ* schizophrenia, *TD* tardive dyskinesia
Respondents provided the burden only for tasks for which they indicated they support the patient.
Patients for whom caregivers were providing care were stratified by the underlying disease if only 1 condition was present or, if > 1 was present, by the condition having the greatest impact. Patients for whom caregivers were providing care with BD+MDD were classified as BD.
