# Supplementary material for: Impact of tardive dyskinesia on patients and caregivers: a survey of caregivers in the United States
Source: J Patient Rep Outcomes. 2023 Nov 28;7:122. doi: 10.1186/s41687-023-00658-9 (PMC10684842; doi:10.1186/s41687-023-00658-9)
Supplement: Supplementary file 4 — Additional file 4: Figure S4. Impact on caregiver daily activities by patient’s underlying condition. [file 41687_2023_658_MOESM4_ESM.pdf]

Figure S4. Impact on caregiver daily activities by patient's underlying condition

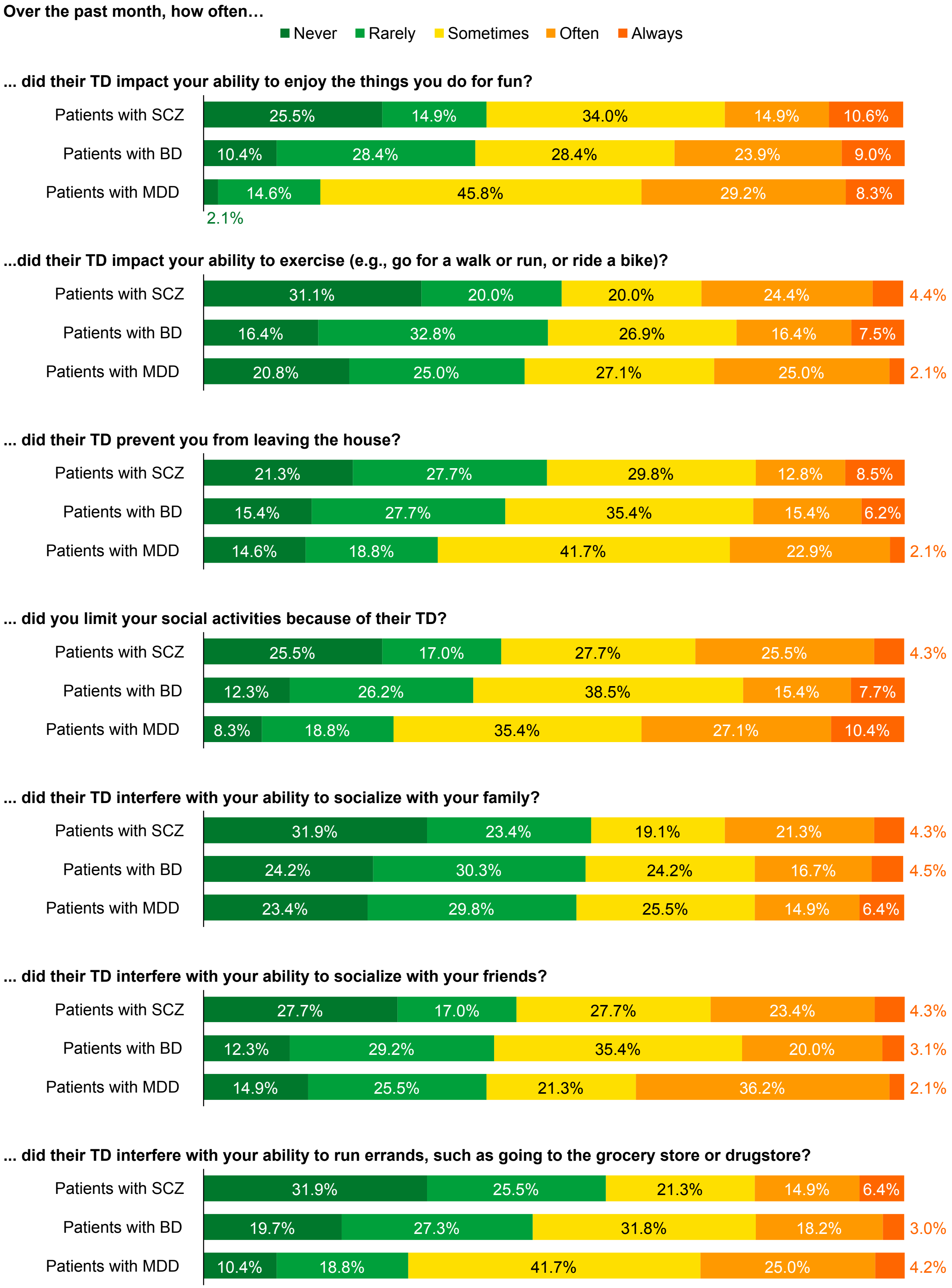

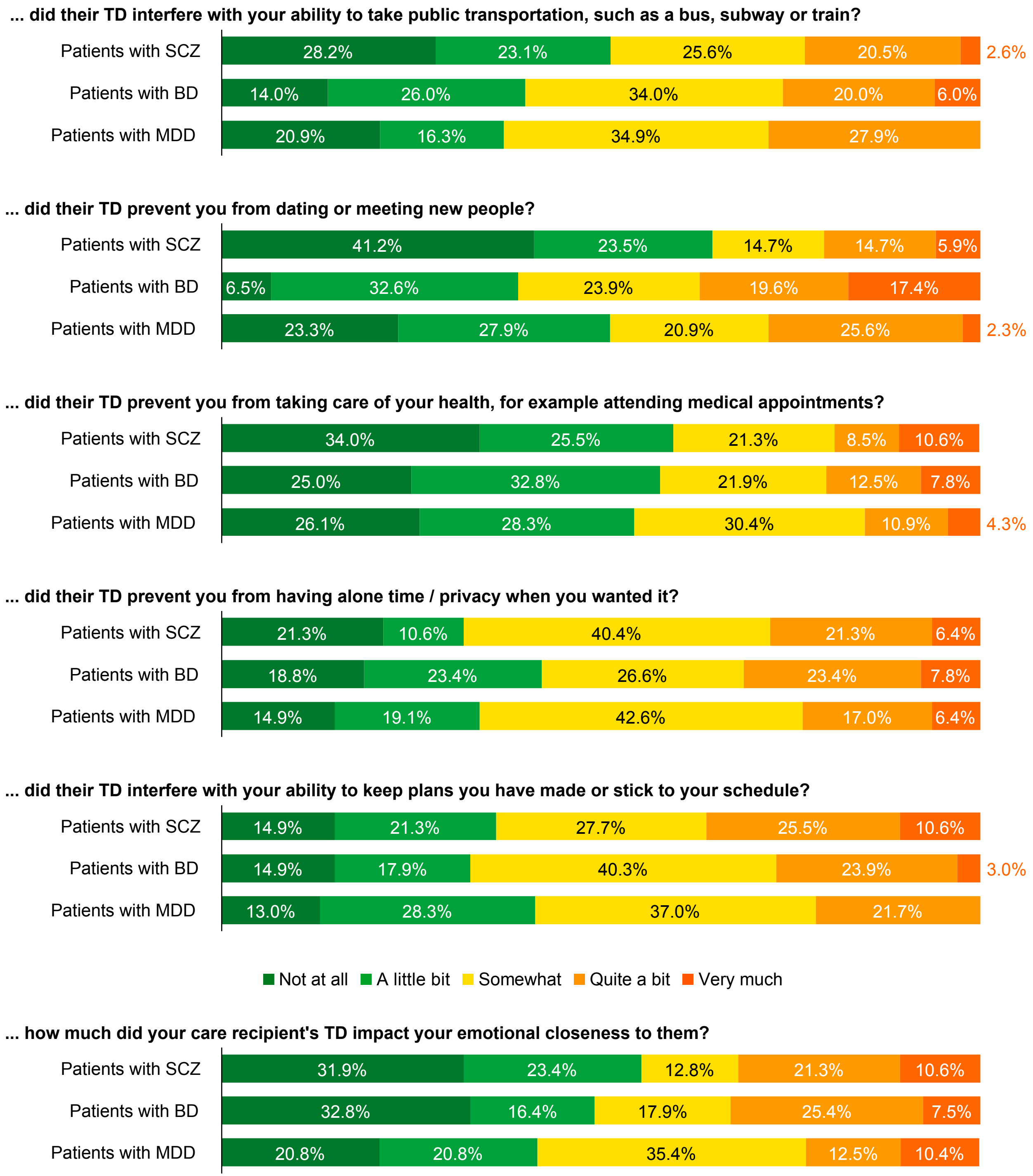

BD bipolar disorder, *MDD* major depressive disorder, *SCZ* schizophrenia, *TD* tardive dyskinesia
